# Supplementary figures and images for: Phylogenetic diversity analysis of shotgun metagenomic reads describes gut microbiome development and treatment effects in the post-weaned pig
Source: PLoS One. 2022 Jun 24;17(6):e0270372. doi: 10.1371/journal.pone.0270372 (PMC9232140; doi:10.1371/journal.pone.0270372)

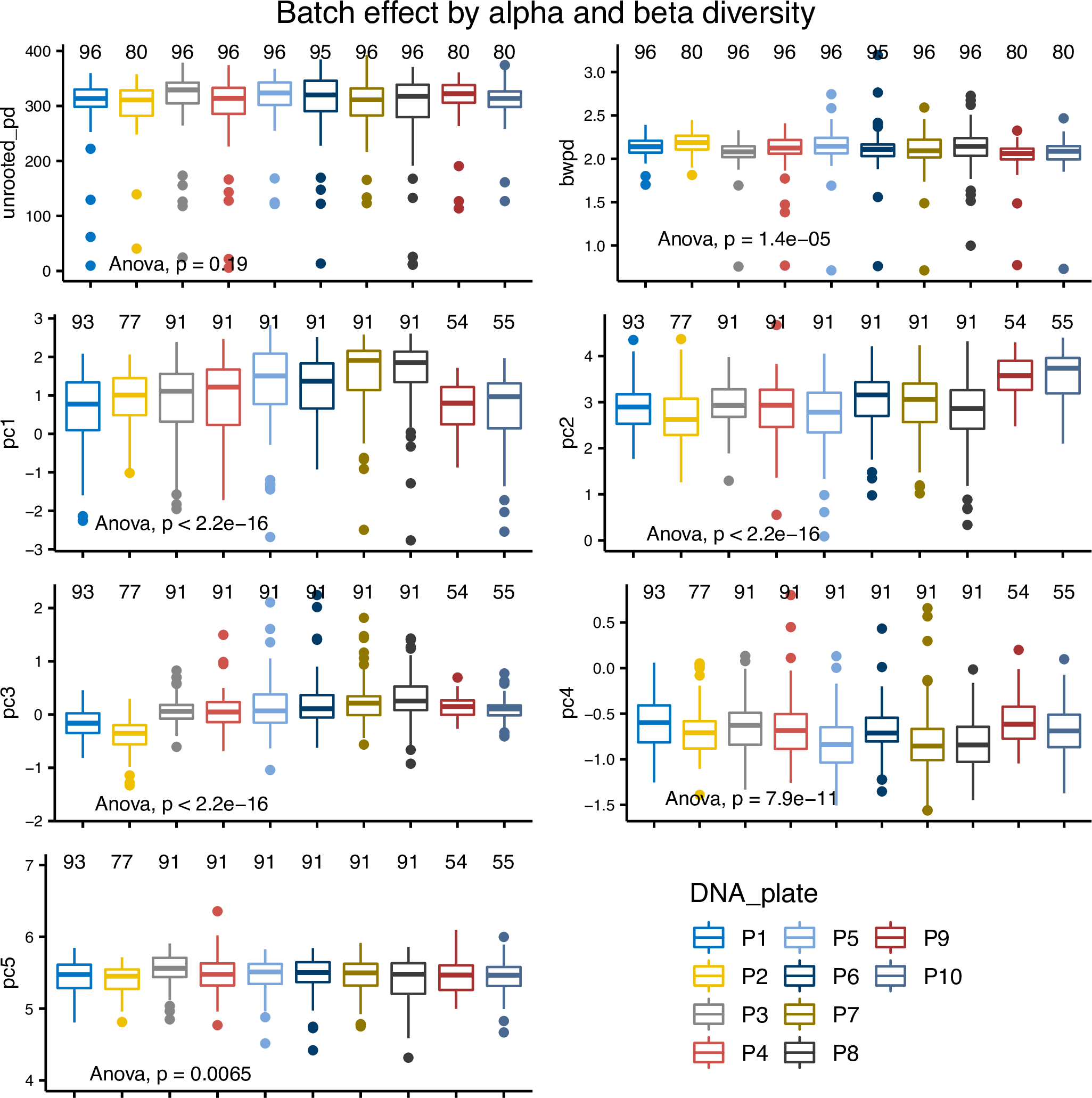

Supplement: S1 Fig — Batch effect by alpha (top two plots) and beta diversity (bottom five plots) before batch effect removal. Samples are grouped by DNA extraction plate. The p values are derived from multiple comparison analysis with ANOVA, indicating equality of the means. Post hoc corrected p values for pairwise comparisons are provided in S1 Table. (TIF) [file pone.0270372.s001.tif]

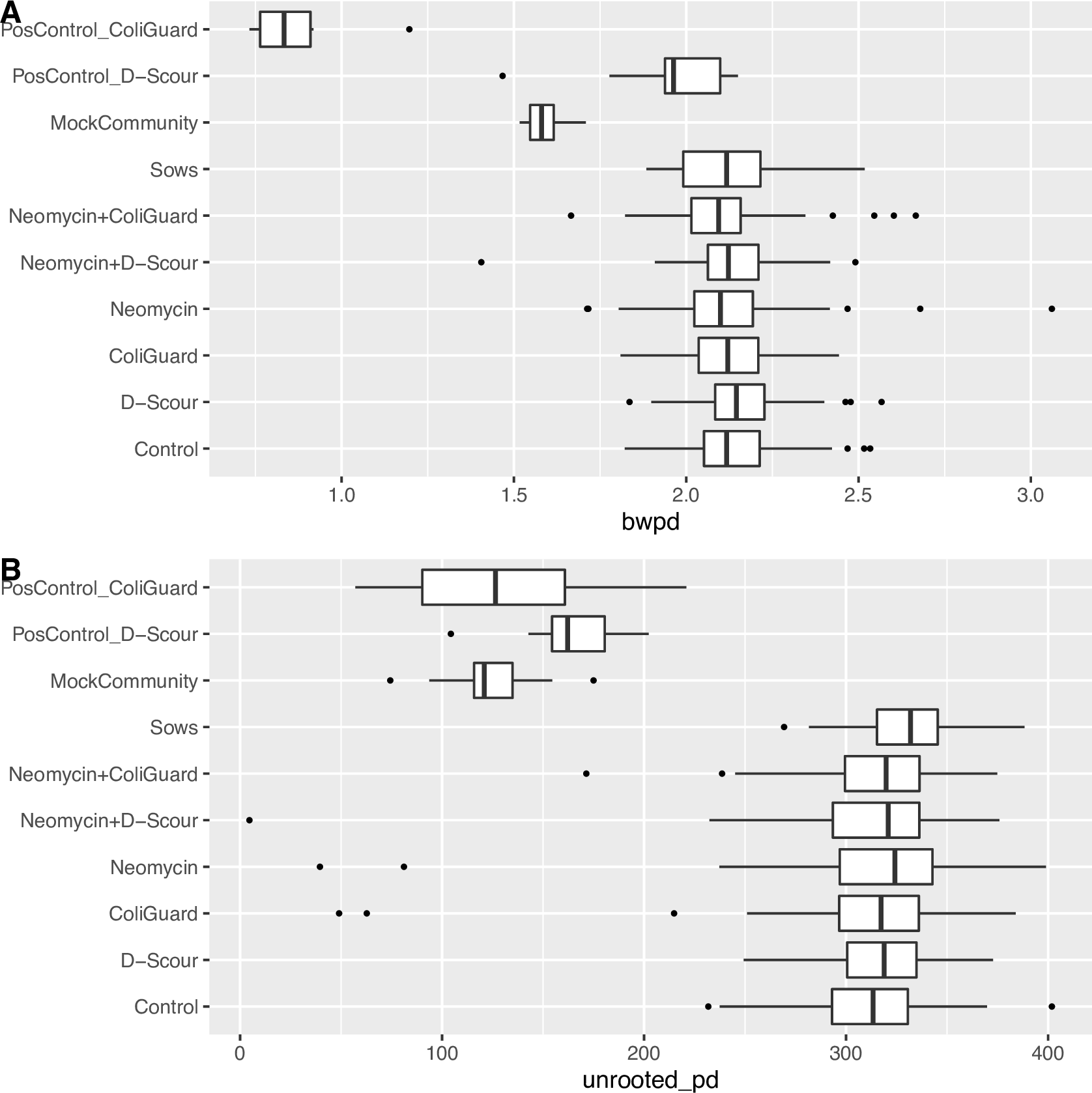

Supplement: S2 Fig — Alpha phylogenetic diversity per cohort from samples across all time points. Balance-weighted phylogenetic diversity (BWPD) (top) (mean±SD: Positive control Mock community: 1.59±0.07; Positive control D-Scour™: 1.94±0.21; Positive control ColiGuard®: 0.86±0.14; Control: 2.13±0.13; D-Scour™: 2.16±0.12; ColiGuard®: 2.12±0.12; neomycin: 2.12±0.16; neomycin+D-Scour™: 2.13±0.13; neomycin+ColiGuard®: 2.10±0.14; sows: 2.12±0.15; all piglet cohorts: 2.13±0.13); Unrooted phylogenetic diversity (bottom) (mean±SD: Positive control Mock community: 123.62±24.41; Positive control D-Scour™: 162.14±28.27; Positive control ColiGuard®: 129.88±50.00; Control: 311.23±29.23; D-Scour™: 316.35±24.99; ColiGuard®: 311.98±42.51; neomycin: 314.81±44.01; neomycin+D-Scour™: 312.66±40.18; neomycin+ColiGuard®: 316.29±31.20; sows: 328.51±24.00; all piglet cohorts: 313.86±36.00). (TIF) [file pone.0270372.s002.tif]

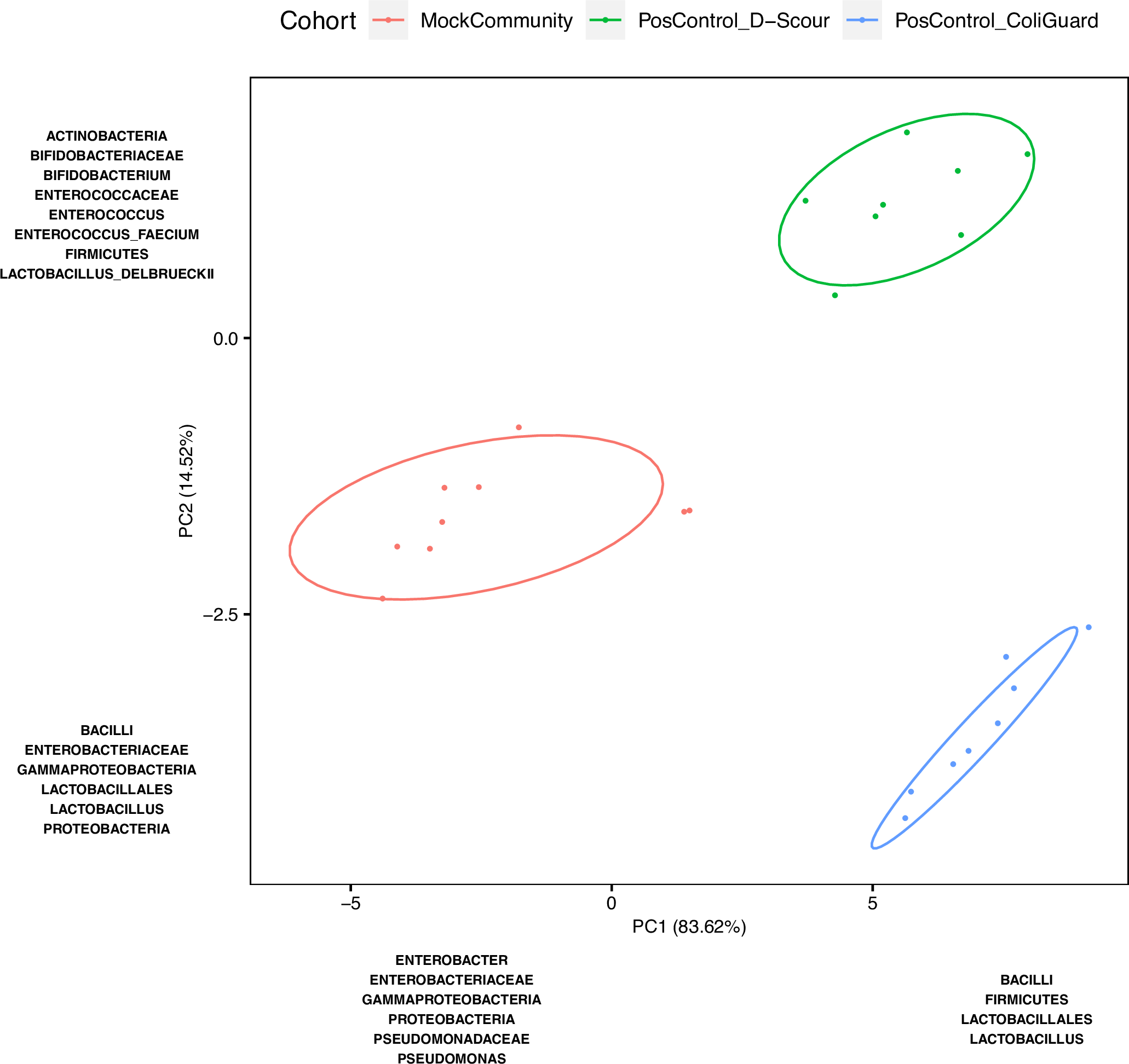

Supplement: S3 Fig — Principal component analysis (PCA) of positive control samples. PCA from edge component analysis with PhyloSift. Distribution of samples on either side of the plot (left versus right; top versus bottom) reflect the lineages that were found to explain the variation. (TIF) [file pone.0270372.s003.tif]

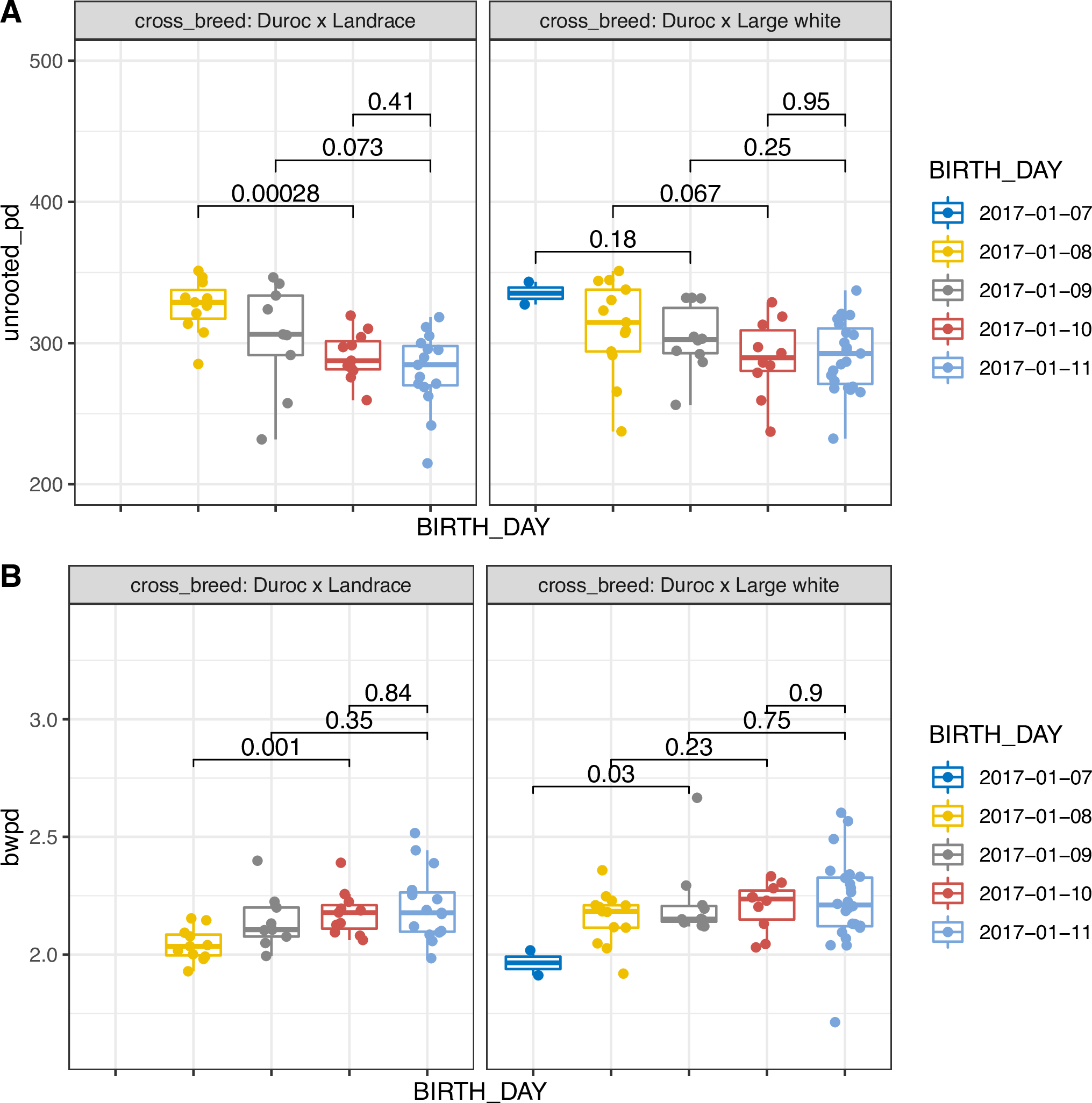

Supplement: S4 Fig — Alpha diversity of samples from the start of the trial (immediately after weaning) grouped by breed and by date of birth. Unrooted phylogenetic diversity (top) and balance-weighted phylogenetic diversity (bottom). P values are derived from Kruskal-Wallis analysis of variance. Piglets of the Duroc × Landrace breed (n = 46) separated significantly by age in unrooted phylogenetic diversity and in BWPD at the start of the trial (t0) (Hommel adjusted p value: unrooted pd = 0.006; BWPD = 0.047). All post hoc corrected p values are provided in S1 Table. (TIF) [file pone.0270372.s004.tif]

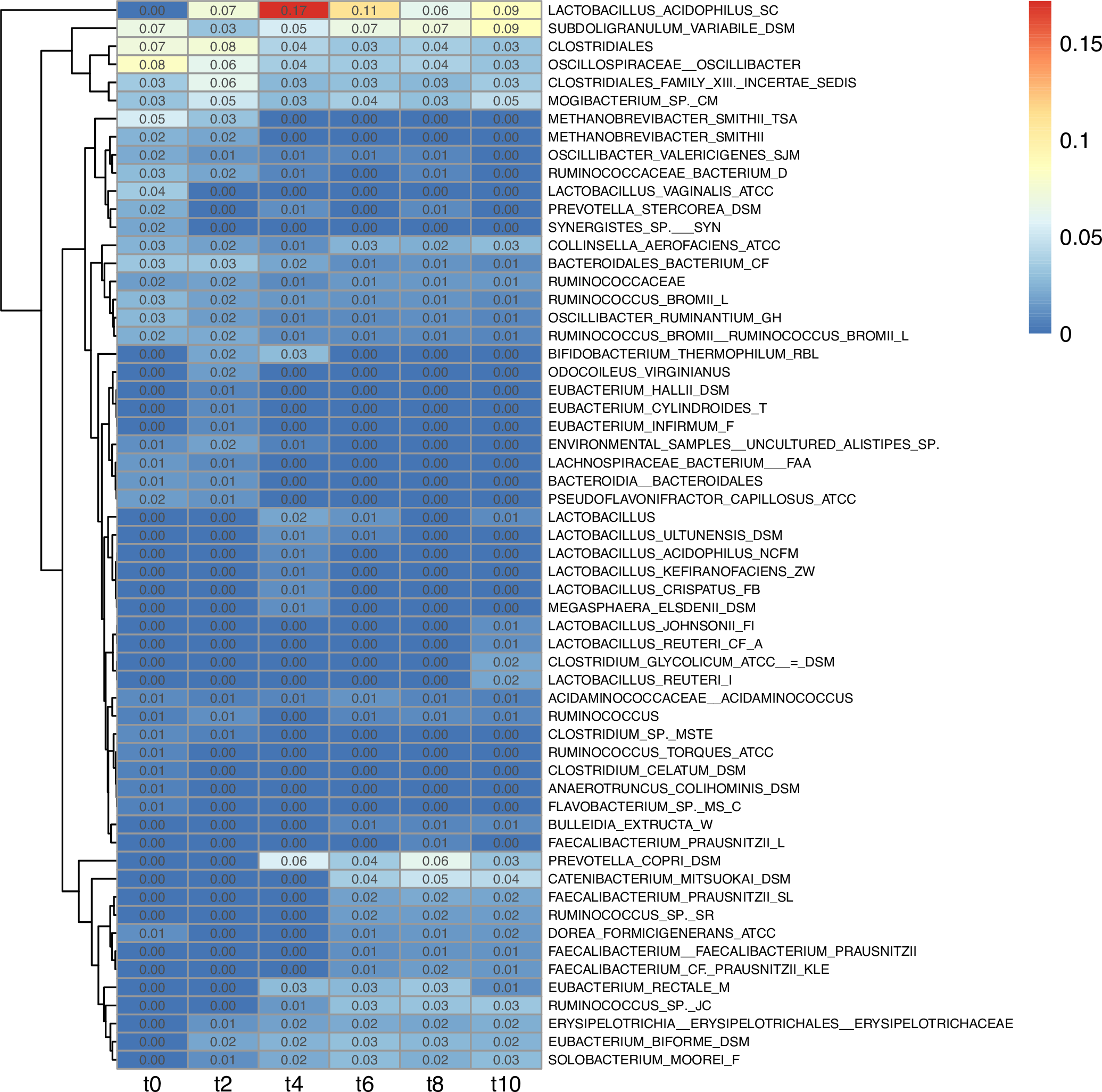

Supplement: S5 Fig — Most abundant lineages within each time point (columns) are obtained from analysis with guppy fat. Guppy fat outputs trees with fattened edges in proportion to the relative abundance of reads place in each lineage. The branch width of the trees, each corresponding to samples from distinct time points, are the entries for this heat map. The distance between each time point is one week. (TIF) [file pone.0270372.s005.tif]

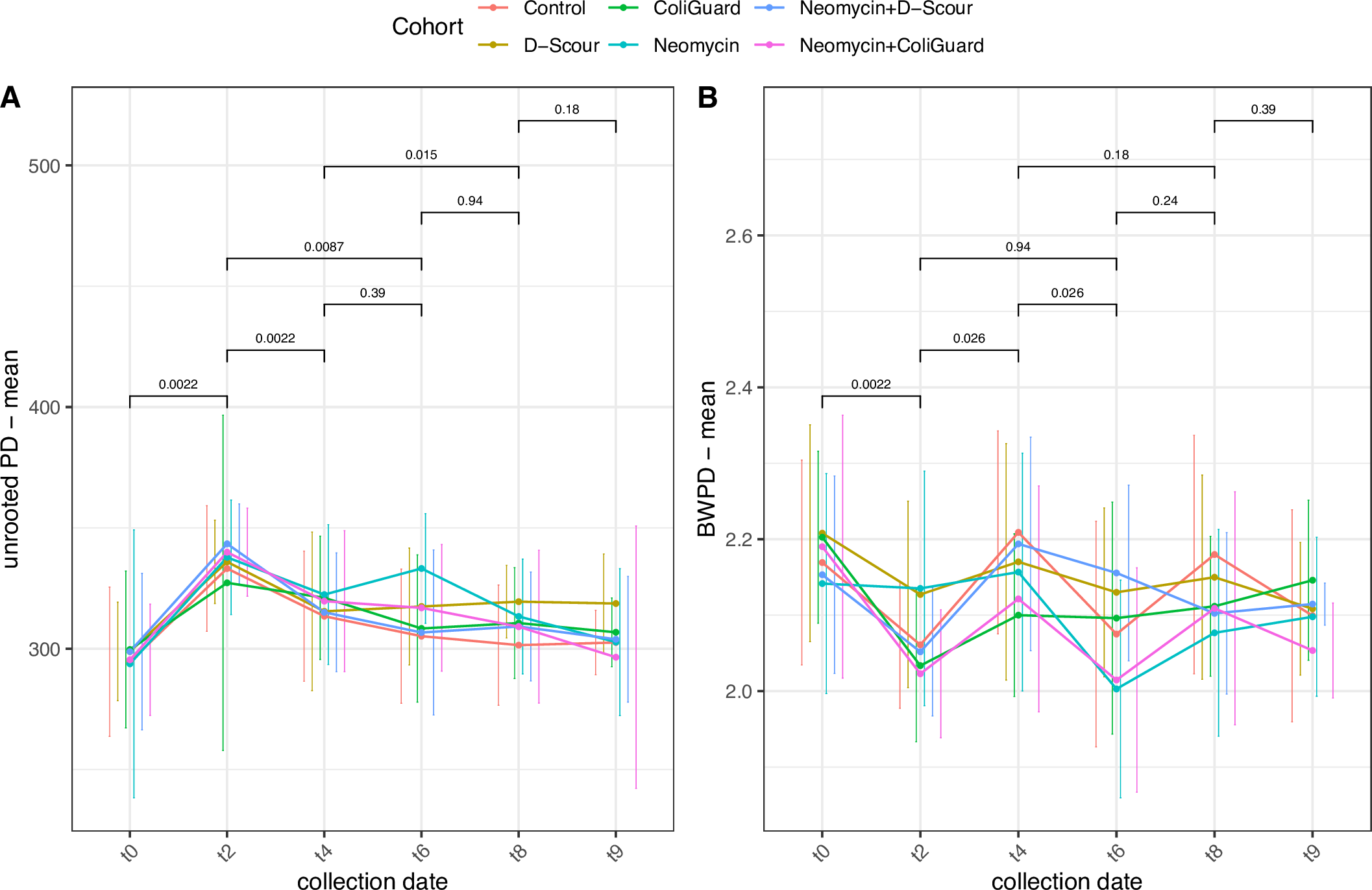

Supplement: S6 Fig — Unrooted phylogenetic diversity (A) and balance-weighted phylogenetic diversity (B) describe richness and evenness, respectively, of alpha phylogenetic diversity for all samples across time, grouped and color coded by cohort. The p values derive from pairwise comparisons of time points of all treatment cohorts. The p values and post hoc corrected p values of time points comparisons for each separate treatment cohort are provided in S1 Table. (TIF) [file pone.0270372.s006.tif]

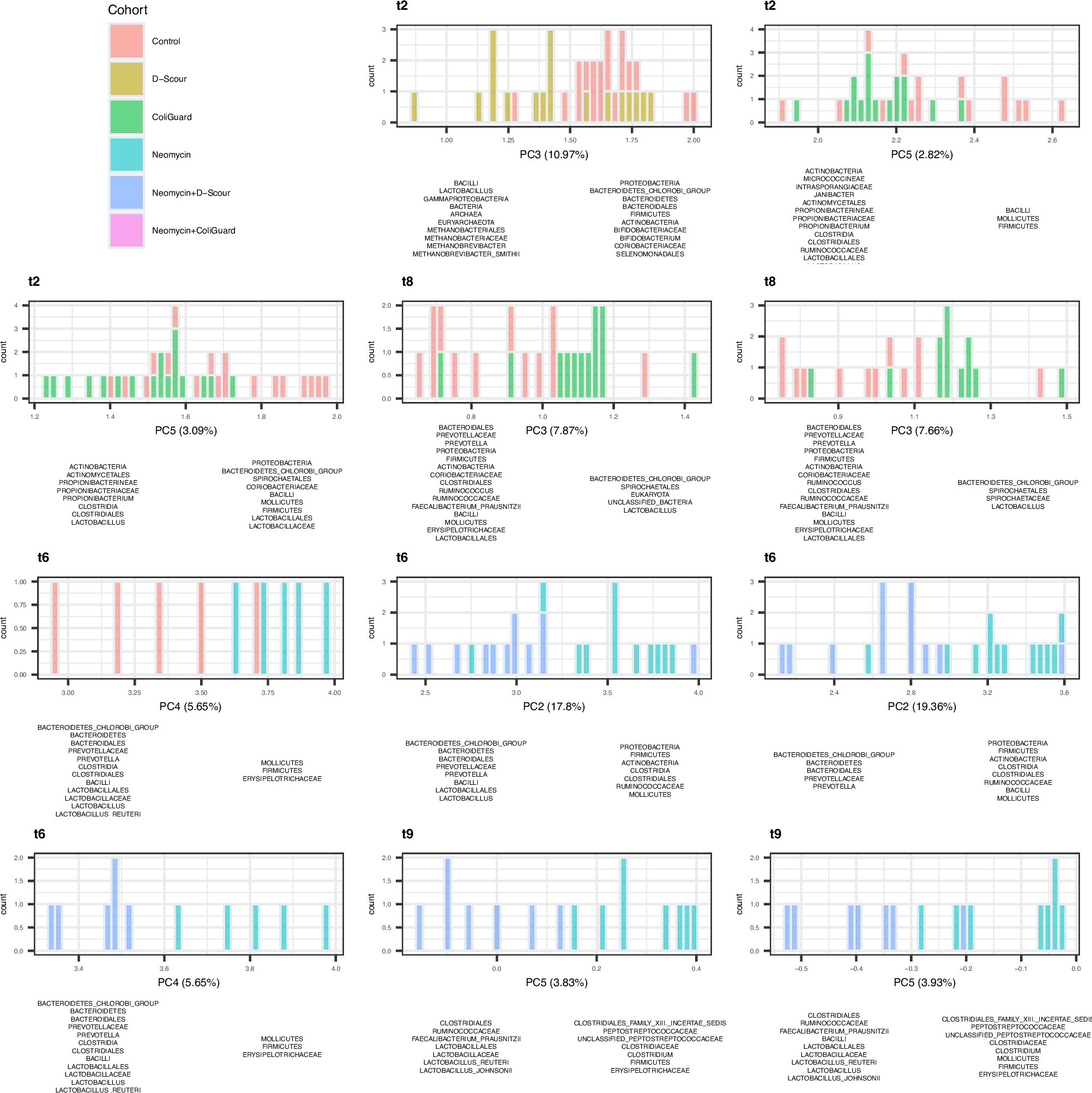

Supplement: S7 Fig — Significance was determined by comparing groups by pairwise t-test and the resulting p values were adjusted with the Bonferroni method. Significance values are provided in S1 Table. The x-axes represent the principal component. As plots are derived from distinct guppy runs, each principal component explains variation to a different extent (percentage specified in parentheses). The number of samples is specified on the y-axis. Distribution of the samples on either side of a plot (left versus right) reflects the lineages that were found to explain the variation. Distributions are color coded by cohort. (TIF) [file pone.0270372.s007.tif]

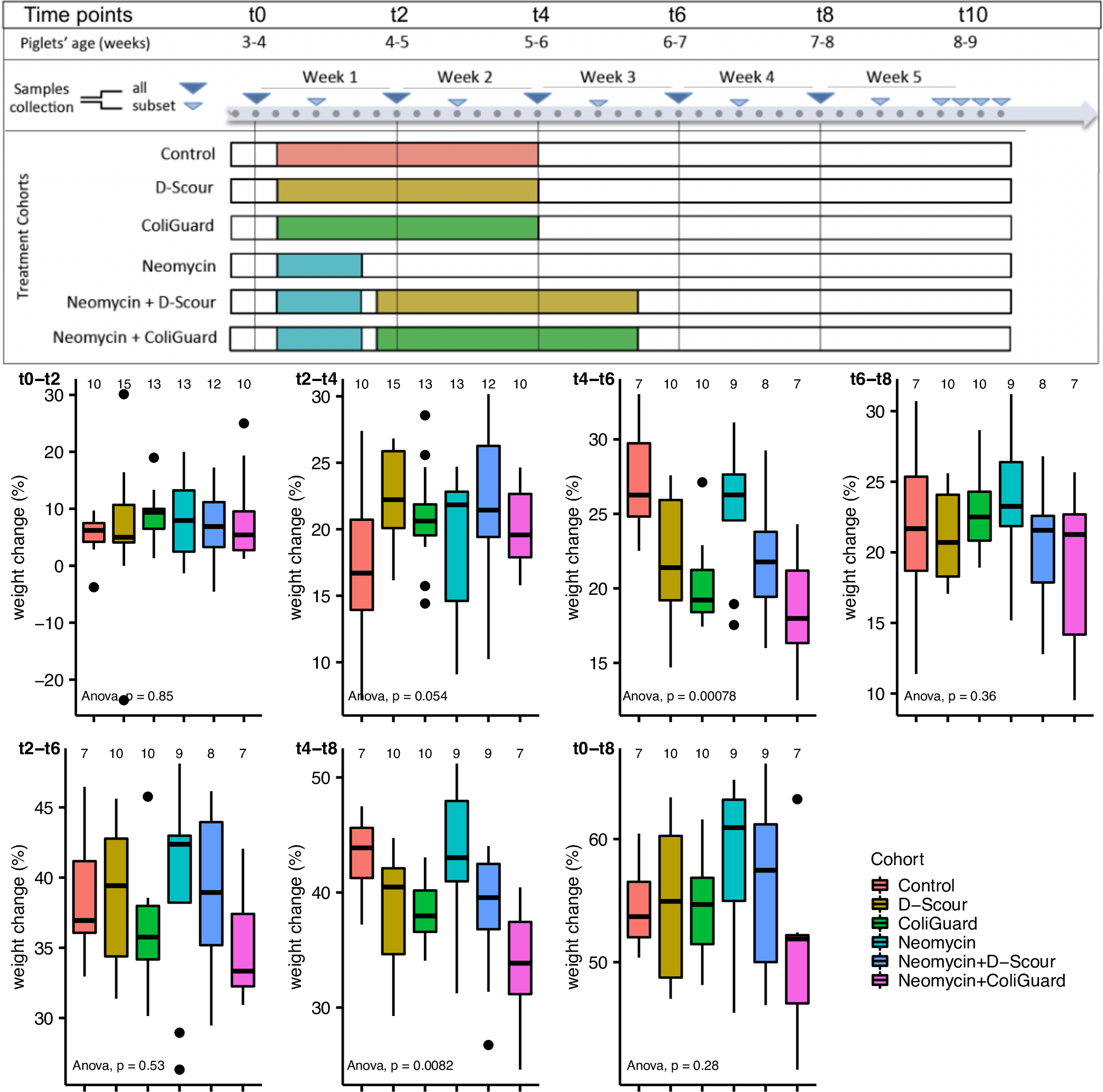

Supplement: S8 Fig — On the y-axis of plots the change in weight gain between time points is provided in percentage. Letters on the top left of each plot indicate the time points compared with one week interval (t0-t2, t2-t4, t6-t8, t8-t10) and with two weeks interval (t2-t6, t4-t8, t0-t8). Pairwise t-test comparisons between cohorts were computed. A significant difference was found between Control and ColiGuard (t4-t6, Tukey adjusted p value = 0.0084), between neomycin and neomycin+ColiGuard (t4-t6, Tukey adjusted p value = 0.0152) and between neomycin and neomycin+ColiGuard (t4-t8, Tukey adjusted p value = 0.011). (TIF) [file pone.0270372.s008.tif]

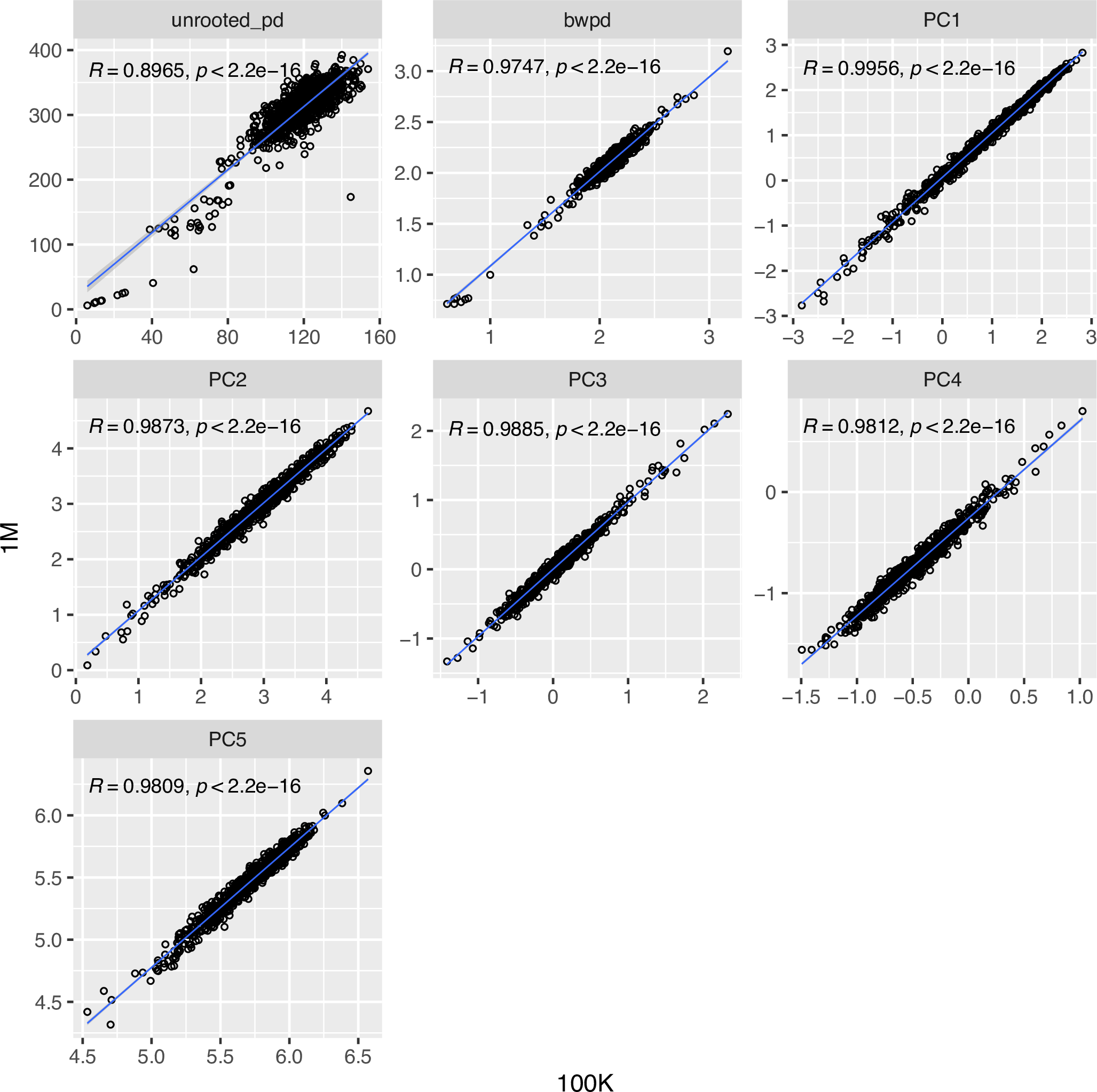

Supplement: S9 Fig — Phylogenetic diversity analysis (alpha and beta) was run using either 100K or 1M reads per sample, corresponding to a 0.6% or 6% of the average sample. We show the Pearson’s correlation of these analyses run using distinct downsampling sizes. Significance of correlations is reported within each plot. All the diversity indices are provided in S1 Table. (TIF) [file pone.0270372.s009.tif]
